# Spectroscopic Study of the Salicyladazine Derivative–UO_2_^2+^ Complex and Its Immobilization to Mesoporous Silica

**DOI:** 10.3390/nano9050688

**Published:** 2019-05-02

**Authors:** Sujin Park, Jaehyeon Park, Ji Ha Lee, Myong Yong Choi, Jong Hwa Jung

**Affiliations:** 1 Department of Chemistry and Research Institute of Natural Sciences, Gyeongsang National University, Jinju 52828, Korea; 0165255562@naver.com (S.P.); parkjae@gnu.ac.kr (J.P.); 2 Department of Chemistry and Biochemistry, The University of Kitakyushu, Hibikino, Kitakyushu 808-0135, Japan

**Keywords:** UO_2_^2+^, salicyladazine, fluorescence, mesoporous silica

## Abstract

Uranyl ion, the most soluble toxic uranium species, is recognized as an important index for monitoring nuclear wastewater quality. The United States Environmental Protection Agency (US EPA) and the World Health Organization (WHO) prescribed 30 ppb as the allowable concentration of uranyl ion in drinking water. This paper reports on a nanohybrid material that can detect uranyl ions spectroscopically and act as a uranyl ion absorbent in an aqueous system. Compound **1**, possessing a salicyladazine core and four acetic acid groups, was synthesized and the spectroscopic properties of its UO_2_^2+^ complex were studied. Compound **1** had a strong blue emission when irradiated with UV light in the absence of UO_2_^2+^ that was quenched in the presence of UO_2_^2+^. According to the Job’s plot, Compound **1** formed a 1:2 complex with UO_2_^2+^. When immobilized onto mesoporous silica, a small dose (0.3 wt %) of this hybrid material could remove 96% of UO_2_^2+^ from 1 mL of a 100-ppb UO_2_^2+^ aqueous solution.

## 1. Introduction

The development of nuclear technology leads to new environmental concerns, such as radiation exposure and accidents resulting therefrom. Of special concern is the uranyl cation (UO_2_^2+^), a highly toxic neurotoxin that is very mutable in biological systems and can cause radioactive poisoning if proper containment rules are violated [1,2,3,4]. Therefore, the development of technologies that can measure the exact amount of UO_2_^2+^ exposed to the environment is an important safety priority.

Several studies on UO_2_^2+^ sensors have been reported to date [5,6,7,8,9,10]. L. S. Natrajan et al. reported a method for detecting UO_2_^2+^ via a unique fluorescence energy transfer process to a water-soluble europium (III) lanthanide complex triggered by UO_2_^2+^ [11]. Yi Lu et al. developed colorimetric uranium sensors based on a UO_2_^2+^-specific DNAzyme and gold nanoparticles using both labeled and label-free methods [12]. Julius Rebek Jr. et al. investigated a tripodal receptor capable of extracting uranyl ion from aqueous solutions. In their system, at a uranyl concentration of 400 ppm, the developed ligand extracted approximately 59% of the UO_2_^2+^ into the organic phase [13].

**Figure 1:**
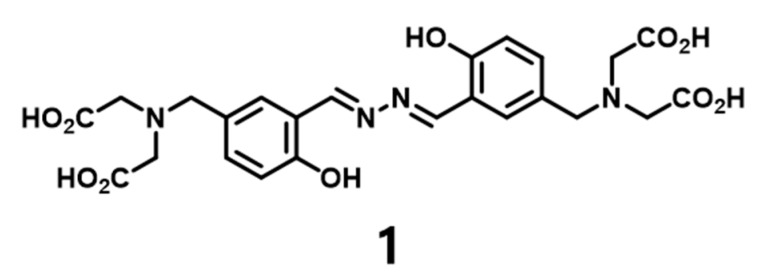
Chemical structure of compound **1**.

While various detection methods for UO_2_^2+^ have been developed based on fluorogenic and colorimetric methods, studies on UO_2_^2+^ adsorbents have received much less attention. We aim to synthesize an adsorbent, or uranyl-capture agent based on an organic–inorganic hybrid material because such compounds tend to have higher stability and controllable homogenous pore sizes. Therefore, we designed and synthesized compound **1** (Figure 1). Compound **1** possesses four acetic acid groups as ligands for UO_2_^2+^. The spectroscopic properties of compound **1** were observed upon adding UO_2_^2+^ via fluorometry, IR spectroscopy, and ^1^H NMR spectroscopy. Furthermore, compound **1** was immobilized to **MPS** (mesoporous silica nanoparticles) to create an adsorbent for UO_2_^2+^. Herein, we report the spectroscopic properties of the compound **1**–UO_2_^2+^ complex and the adsorption capacity of mesoporous silica nanoparticles loaded with compound **1** for UO_2_^2+^ capture. 

## 2. Materials and Methods

### 2.1. Reagents and Instruments

All reagents were purchased from Sigma-Aldrich (Buchs, Switzerland) and Tokyo chemical industry (Fukaya, Japan). The solvent was purchased from Samchun Pure Chemicals (Pyeongkaek, Korea) and used with further purification. ^1^H and ^13^C NMR spectra were obtained with a Bruker DRX 300 apparatus (Rheinstetten, Germany). The IR spectra were measured on a Shimadzu FT-IR 8400S instrument (Kyoto, Japan) by KBr pellet method in the range of 4000−1000 cm^−1^. A JEOL JMS-700 mass spectrometer (Kyoto, Japan) was used to obtain the mass spectra. The UV−vis absorption and fluorescence spectra were obtained at 298 K with a Thermo Evolution 600 spectrophotometer (Waltham, MA, USA) and a RF-5301PC spectrophotometer (Kyoto, Japan), respectively. A PerkinElmer 2400 series (Waltham, MA, USA) was employed for the elemental analyses. The quantitative analysis was performed using ICP-DRC-MS (ELAN DRC II, PerkinElmer, Waltham, MA, USA). The morphological images were observed using a TEM (TECNAI G2 F30, FEI, Hillsboro, OR, USA).

### 2.2. Synthesis of Compound **1**

The compounds **1**–**4** were synthesized according to the reported method (Scheme S1) [14,15]. Compound **2** (2.56 mg, 0.4 mmol) was dissolved in THF (10 mL), followed by the addition of sodium hydroxide solution (10 mL, 0.8 M). The mixture was stirred at room temperature for 2 h. After completion of the reaction, the mixture was added to aq HCl solution (1 wt %) to give yellow precipitation, which was filtered off and dried under vacuum to yield compound **1** (121 mg, 57%). IR (KBr pellet) cm^−1^ 3424, 3014, 1728, 1495 1629, 1389, 1277, 1164; ^1^H NMR (300 MHz, DMSO-*d*_6_) δ 12.25 (s, 4H), 11.07 (s, 2H), 8.98 (s, 2H), 7.63 (d, *J* = 2.2 Hz, 2H), 7.40 (dd, *J* = 8.5, 2.2 Hz, 2H), 6.96 (d, *J* = 8.4 Hz, 2H), 3.78 (s, 4H), 3.42 (s, 8H); ^13^C NMR (75 MHz, DMSO-*d*_6_) δ 172.74, 162.82, 158.30, 134.37, 131.08, 130.12, 118.33, 116.99, 56.80, 53.96.; ESI-MS: calculated for C_24_H_26_N_4_O_10_, [M − H]^−^ 529.16; found, 529.15; Anal. calcd for C_24_H_26_N_4_O_10_: C, 54.34; H, 4.94; N, 10.56; found: C 54.31, H 4.97, N 10.51.

### 2.3. Preparation of **MPS**

The **MPS** was synthesized according to the reported method [16]. 8.2 g of (1-Hexadecyl) trimethyl-ammonium bromide was dissolved in H_2_O (600 mL). After stirring for 10 min, 1.6 mL of triethanolamine was added and the reaction mixture was heated. When the reaction temperature reached 80 °C, TEOS (tetra ethyl ortho silicate) (60 mL) was added and stirred for 1 h. The solvent of the reaction was removed by rotary evaporator and the resulting solid (including some water) was heated at 500 °C for 5 h by using a furnace.

### 2.4. Preparation of **MPS-1**

0.1 g of compound **1** and 1 g of **MPS** in of acetonitrile (20 mL) were stirred for 10 min. The reaction mixture was refluxed at 80 °C for 24 h. After the reaction mixture was cooled to room temperature, the solid product was filtered and washed with 200 mL acetonitrile.

### 2.5. Photophysical Studies

The UV−vis absorption and fluorescence spectra were determined over the range 200−800 nm. The samples were prepared by dispersion in H_2_O solution. The concentration of standard UO_2_^2+^ solution was 100 ppb.

### 2.6. NMR Measurement

Compound **1** (2.65 mg, 0.005 mmol) and uranyl acetate (8.48 mg, 0.02 mmol) were dissolved in 0.5 mL and 0.1 mL of of DMSO-*d*_6_ (0.5 mL) in DMSO-*d*_6_ (0.1 mL), respectively. To NMR titration, the different amount of the uranyl acetate solution (12.5 μL, 25 μL, 37.5 μL, 50 μL) was added to compound **1** of DMSO-*d*_6_ (0.5 mL).

### 2.7. ICP-MS

**MPS-1** (1 mg, 3 mg, and 5 mg) were dispersed in aqueous solution containing UO_2_^2+^, Na^+^, Mg^2+^, Ca^2^^+^, Cu^2^^+^, Ag^+^, Co^2+^, Ni^2+^, Mn^2+^ and Pb^2^^+^ (100 ppb) for 10 min. Mixture was added to H_2_O (4 mL). The mixture solution was centrifuged and the supernatant solution was filtrated with syringe filter (PTFE, 0.45 μm). The collected solution was measured 3 times by ICP-MS.

## 3. Results and Discussion

### 3.1. Spectroscopic Properties of Complex **1** with UO_2_^2+^

Binding between UO_2_^2+^ and specific ligands, such as cyclic peptides [17,18], porphyrins [19,20], and naphthobipyrrole [21,22], is well known. We prepared a salicyladazine derivative as a ligand for UO_2_^2+^. The salicyladazine derivative was synthesized starting from 2-hydroxybenzaldehyde. The diethyl 2,2′-azanediyldiacetate groups were designed on both sides of the compound to create a symmetric structure. As the final step, hydrochloric acid treatment of the precursor yielded the desired compound **1**. Compound **1** contains the acetic acid end group (–CH_2_COOH) to form the binding site for UO_2_^2+^ and was characterized via FT IR, ^1^H and ^13^C NMR, mass spectrometry, and elemental analysis (Figures S1, S2 and S3 in Supplementary Materials).

**Figure 2:**
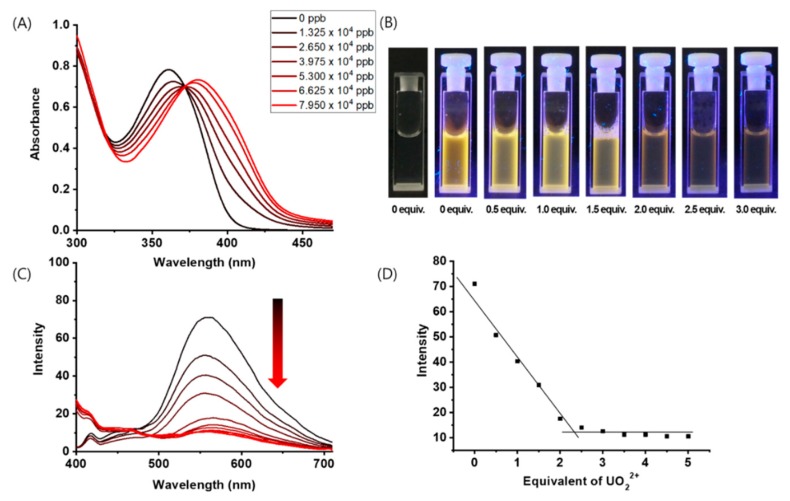
(**A**) UV–vis spectra of compound **1** (2.65 × 10^4^ ppb) in DMSO/H_2_O (1:99 *v/v*) containing various amounts of UO_2_^2+^. (**B**) Photographed cuvettes from (**A**) under UV light irradiation. (**C**) Fluorescence spectra of compound **1** (2.65 × 10^4^ ppb) in DMSO/H_2_O (1:99 *v/v*) containing various amounts of UO_2_^2+^ (0–5 equivalents). (**D**) Plot of fluorescence intensity of (**C**) vs. amount of UO_2_^2+^.

UV–vis spectroscopy was performed to confirm that a coordination bond between compound **1** and UO_2_^2+^ resulted in a colorimetric change. Compound **1** was dissolved in an aqueous solution containing 1% of DMSO, and the UV–vis absorption spectrum was measured (Figure 2A). Before adding UO_2_^2+^, the π–π * absorption band of compound **1** appeared at around 300–350 nm. When UO_2_^2+^ (from 0.5 to 3 equivalents in 0.5 equivalent steps) was added to compound **1**, the absorption peak intensities at 300 and 350 nm decreased until up to 2 equivalents of UO_2_^2+^ were added. At 2.5 or more equivalents of UO_2_^2+^, the ligand-to-metal charge transfer absorption wavelength between compound **1** and UO_2_^2+^ was observed at around 365–380 nm [23].

Figure 2B shows the photographs of the cuvettes used when UO_2_^2+^ was added to compound **1** and irradiated under UV light. The change in fluorescence after more than 2 equivalents of UO_2_^2+^ were added was visible to the naked eye. Compound **1** yielded an emission wavelength around 560 nm (excitation = 365 nm). When 0.5 equivalents of UO_2_^2+^ were added to compound **1**, the fluorescence intensity decreased. The decrease in fluorescence was noticeable from 0.5 to 2 equivalents of UO_2_^2+^; however, the fluorescence intensity remained constant thereafter (Figure 2C). Figure 2D presents a plot of the fluorescence intensity vs. amount of UO_2_^2+^ added. To determine the stoichiometric ratio between compound **1** and UO_2_^2+^, we constructed a Job’s plot using the fluorescence data and found a 1:2 binding ratio for compound **1**:UO_2_^2+^ (Figure S4 ).

**Figure 3:**
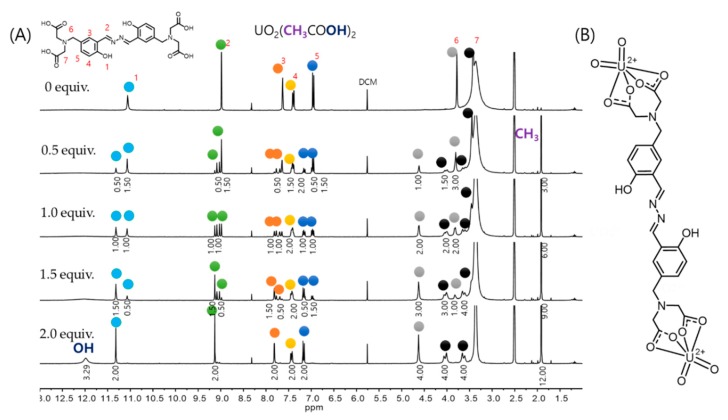
(**A**) ^1^H nuclear magnetic resonance (NMR) spectra of compound **1** (10 mM) in DMSO-*d*_6_ containing various equivalents of uranyl acetate. (**B**) Proposed structure of complex **1** with UO_2_^2+^.

To investigate the chemical interactions between compound **1** and UO_2_^2+^, we used nuclear magnetic resonance (NMR) spectroscopy. We measured the ^1^H NMR signal of compound **1** in DMSO-*d*_6_ while increasing the UO_2_^2+^ content (Figure 3). When 0.5 equivalent of UO_2_^2+^ was added to compound **1** in DMSO-*d*_6_, the proton peak (aromatic OH: 11.08 ppm) of compound **1** decreased and multiple new peaks were observed. When we added 1 equivalent of UO_2_^2+^, the ratio of the proton peaks of compound **1** and the resulting complex was 1:1. Because compound **1** has C2 symmetry, coordination of UO_2_^2+^ occurs on one side of compound **1** (bound to two acetic acid ligands) and no coordination occurs on the other side of compound **1**. Upon additional UO_2_^2+^ input (over 2 equivalents), all the peaks representing free compound **1** disappeared entirely. Therefore, compound **1** has a binding capacity of two UO_2_^2+^ molecules in DMSO-*d*_6_ (forms a 1:2 complex). This result is consistent with the Job’s plot.

The carbonyl oxygen (C=O) of the end group of acetic acids (–CH_2_COOH) in compound **1** is well known to bind strongly to a radionuclide ion such as UO_2_^2+^ by supplying electrons [23,24]. IR spectroscopy was used to classify the complex formation of compound **1** with UO_2_^2+^. As shown in Figure S5, the oxygen of the carboxylic acid carbonyl (C=O) in compound **1** before the addition of UO_2_^2+^ produced a peak at 1728 cm^−1^, whereas the C=O peak after the addition of UO_2_^2+^ was shifted to 1557 cm^−1^. This shift to a lower wavenumber is indicative of the C=O in compound **1** providing electrons in a dative bond to UO_2_^2+^ and confirms that the carboxylic acid groups are employed in complex formation with UO_2_^2+^.

### 3.2. Immobilization of Compound **1** to Mesoporous Silica Nanoparticles

**Figure 4:**
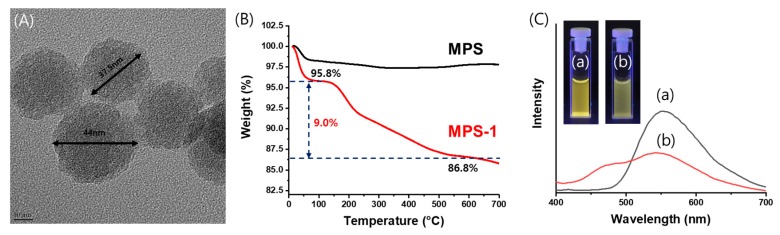
(**A**) Transmission electron microscopy (TEM) image of mesoporous silica (**MPS-1**) and (**B**) thermogravimetry analysis (TGA) thermogram of **MPS** (black) and **MPS-1** (red). (**C**) The photograph and the Fluorescence spectra of (**a**) **MPS-1** (2 mg) in water (2 mL) and (**b**) **MPS-1** (2 mg) in 100 ppb UO_2_^2+^ solution (2 mL).

The morphology of mesoporous silica nanoparticle immobilized with **1** (**MPS-1**) was observed via transmission electron microscopy (TEM). The TEM image of **MPS-1** revealed a spherical structure with a narrow size distribution (circa 40 nm) (Figure 4A). Thermogravimetry analysis (TGA) was performed to determine the amount of compound **1** immobilized onto **MPS-1** (Figure 4B). At approximately 150 °C, the weight of **MPS-1** decreased by 4.2%. This mass reduction was attributed to moisture. At ~500 °C, compound **1** was pyrolyzed and the weight of **MPS-1** decreased to 86.8% (Figure S6). Thus, the amount of compound **1** introduced into **MPS-1** was 9% by weight (Figure 4B). Figure S7 presents the IR spectra of **MPS** and **MPS-1**; a C-H vibration peak of 2940 cm^−1^ was confirmed. This further supported the presence of compound **1** on the surface of the mesoporous silica nanoparticles. Under UV light irradiation, the filtered silica nanoparticles fluoresced blue, indicating that compound **1** was present on the mesoporous silica nanoparticle surface. Fluorescence spectra of **MPS-1** (2 mg) in water (2 mL) and **MPS-1** (2 mg) in 100 ppb UO_2_^2+^ solution (2 mL) were also measured. (Figure 4C). In 3.5% NaCl aqueous solution of 2mL, we measured the fluorescence spectra of **MPS-1** (2 mg) and **MPS-1** (2 mg) in 100 ppb UO_2_^2+^ solution, respectively (Figure S8). Fluorescence changes of **MPS-1** in the present of 100 ppb UO_2_^2+^ in water or 3.5% NaCl aqueous solution were shown similar results. This means that **MPS-1** can bind UO_2_^2+^ not only in water but also NaCl aqueous solution.

### 3.3. The Adsorption Capacity of **MPS-1** for UO_2_^2+^

**Figure 5:**
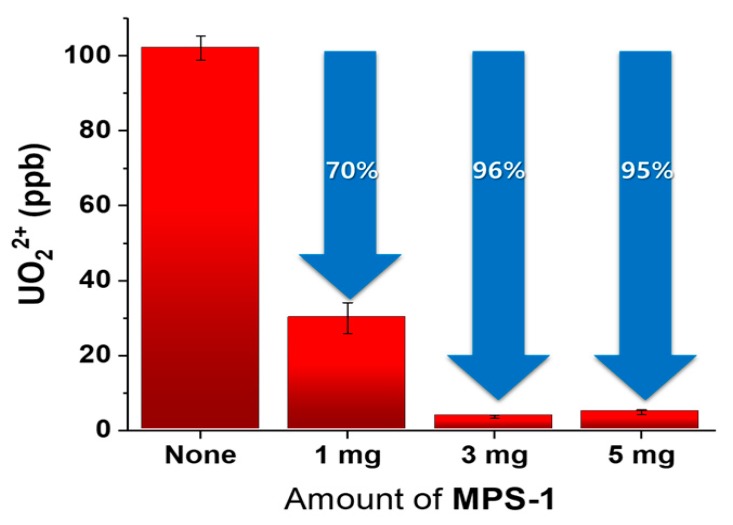
Result of UO_2_^2+^ adsorption of **MPS-1** in 100 ppb UO_2_^2+^ solution.

The United States Environmental Protection Agency (US EPA) and the World Health Organization (WHO) have prescribed safe limits of UO_2_^2+^ in drinking water at 30 ppb [25,26]. The adsorption capacity of **MPS-1** was tested by adding 1, 3, and 5 mg of **MPS-1** to 1 mL of 100 ppb UO_2_^2+^ solution. After standing for 10 min, the solution was filtered through a 0.45-μm syringe filler and the UO_2_^2+^ levels were determined by ICP-MS (experiment performed in triplicate). Calibration curves were obtained with dilute UO_2_^2+^ solutions (0.1, 1, 10, 50, and 100 ng/L), and the linearity of the calibration curve was confirmed (correlation coefficient was 0.9974) (Figure S9). Figure 5 and Table S1 present the results of the UO_2_^2+^ adsorption experiment using various amounts of **MPS-1**. The percentage of UO_2_^2+^ removed was 70%, 96%, and 95% for 1, 3, and 5 mg of **MPS-1**, respectively (RSD values all less than 15%). 1 mg of **MPS-1** was not sufficient for absorbing 100 ppb UO_2_^2+^. Using 3 or 5 mg of **MPS-1** removed 95% or more of the UO_2_^2+^; there was no statistically significant difference between the two absorbent dose amounts. In conclusion, **MPS-1** (even a small amount: 0.3 wt %) could reduce 100 ppb of UO_2_^2+^ <5 ppb of UO_2_^2+^; this result would satisfy both EPA and WHO drinking water standards.

### 3.4. Adsorption of UO_2_^2+^ and Other Cations onto **MPS-1**

The elemental analysis of **MPS**, **MPS-1**, and UO_2_^2+^-adsorbed **MPS-1** was performed via TEM dispersive X-ray spectroscopy (EDX) (Figure S10). Nitrogen was observed in the EDX spectrum of **MPS-1**, thus providing evidence for the presence of compound **1** in **MPS-1**. In the EDX spectrum of UO_2_^2+^-adsorbed **MPS-1**, uranium was detected. This confirms our rationale in designing this adsorbent: UO_2_^2+^ was bound to the compound **1** attached onto the surface of **MPS**.

We also confirmed the adsorption capacity of **MPS-1** (5 mg) for other metal ions, such as Na^+^, Mg^2+^, Ca^2^^+^, Cu^2^^+^, Ag^+^, Co^2+^, Ni^2+^, Mn^2+^, and Pb^2^^+^ (100 ppb) under the same conditions. Among the metal ions tested, 42.3% of Ca^2+^ was adsorbed onto the surface of **MPS-1**; for the remaining metal ions, <30% were adsorbed (Table S2). These findings suggest that **MPS-1** would be useful as an adsorbent for UO_2_^2+^.

## 4. Conclusions

We synthesized the salicyladazine-based compound **1**, designed to be a uranyl ion capture ligand. Compound **1** formed a 1:2 complex with UO_2_^2+^ as confirmed by the Job’s plot. A fluorescence change was observed when UO_2_^2+^ was bound to compound **1**. IR and NMR measurements were performed to identify compound **1** and the two UO_2_^2+^ coordination sites. Compound **1** was immobilized into mesoporous silica (**MPS-1**); the resulting sorbent could remove 96% of the UO_2_^2+^ from 1 mL of a 100-ppb UO_2_^2+^ aqueous solution. A material was successfully developed that was capable of simultaneously absorbing uranyl ions and detecting their presence by fluorescence. We believe that this organic–inorganic hybrid material paradigm for detecting UO_2_^2+^ will have a broad impact for the study on porous materials and their application.

## Supplementary Materials

The following are available online at https://www.mdpi.com/2079-4991/9/5/688/s1. Scheme S1. Synthesis route of compound **1**; Figure S1. FT-IR spectrum of compound **1**; Figure S2. ^1^H NMR spectrum of compound **1** in DMSO-d_6_; Figure S3. ^13^C NMR spectrum of compound **1** in DMSO-d_6_; Figure S4. Job’s plot for complex formed between compound **1** and UO_2_^2+^; Figure S5. FT-IR spectra of **1** and **1** with UO_2_^2+^; Figure S6. TGA thermogram of compound **1**; Figure S7. FT-IR spectra of (A) **MPS** and (B) **MPS-1**; Figure S8. Fluorescence spectra of (a) **MPS-1** (2 mg) in 3.5% NaCl solution (2mL) and (b) **MPS-1** (2 mg) with UO_2_^2+^ solution (100 ppb) in 3.5% NaCl (2 mL); Figure S9. Linear equation of various concentrations of UO_2_^2+^; Figure S10. TEM EDX mapping of (A) **MPS** (B) **MPS-1** and (C) **MPS-1** with UO_2_^2+^; Table S1. Adsorption Capacities of **MPS-1** for UO_2_^2+^ (100 ppb) solution; Table S2. Adsorption Capacities of **MPS-1** (5 mg) with various metal ions (100 ppb) solution.
