# Supplementary material for: Spectroscopic Study of the Salicyladazine Derivative–UO22+ Complex and Its Immobilization to Mesoporous Silica
Source: Nanomaterials (Basel). 2019 May 2;9(5):688. doi: 10.3390/nano9050688 (PMC6566226; doi:10.3390/nano9050688)
Supplement: Supplementary file 1 [file nanomaterials-09-00688-s001.pdf]

Supplementary Materials

Spectroscopic Study of the Salicyladazine Derivative–UO_2^{2+} Complex and Its Immobilization to Mesoporous Silica

Sujin Park [1], Jaehyeon Park [1], Ji Ha Lee [2,*], Myong Yong Choi [1,*] and Jong Hwa Jung [1,*]

[1] Department of Chemistry and Research Institute of Natural Sciences, Gyeongsang National University, Jinju 52828, Korea; 0165255562@naver.com (S.P.); parkjae@gnu.ac.kr (J.P.)

[2] Department of Chemistry and Biochemistry, The University of Kitakyushu, Hibikino, Kitakyushu 808-0135, Japan

* Correspondence: l-ji@kitakyu-u.ac.jp (J.H.L.); mychoi@gnu.ac.kr (M.Y.C.); jonghwa@gnu.ac.kr (J.H.J.); Tel.: +82-55-772-1488 (J.H.J.)

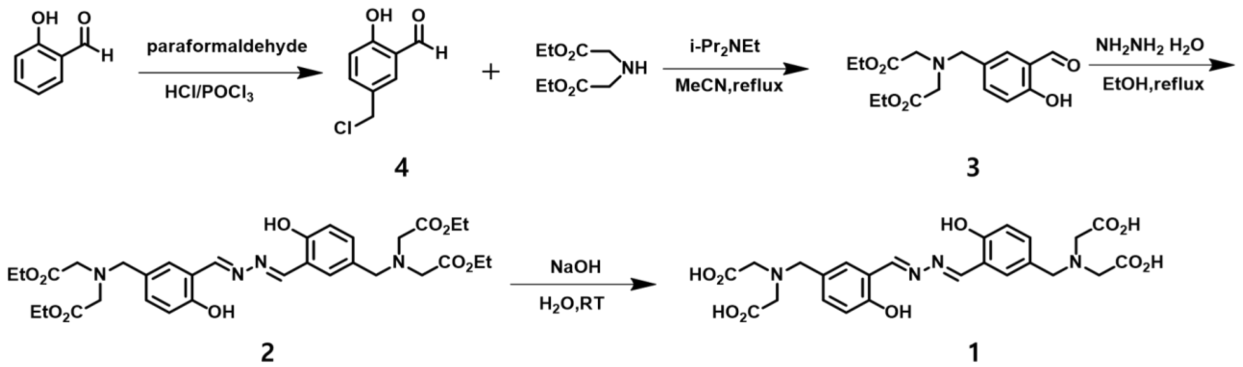

Scheme S1. Synthesis route of compound **1**.

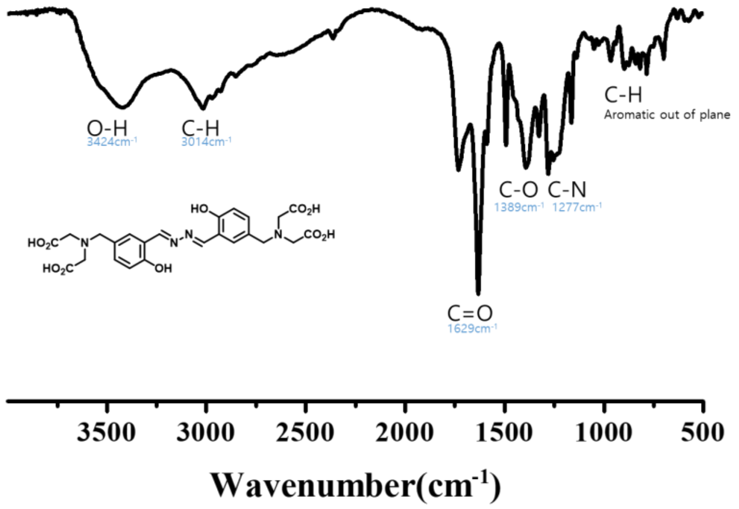

Figure S1. FT-IR spectrum of compound **1**.

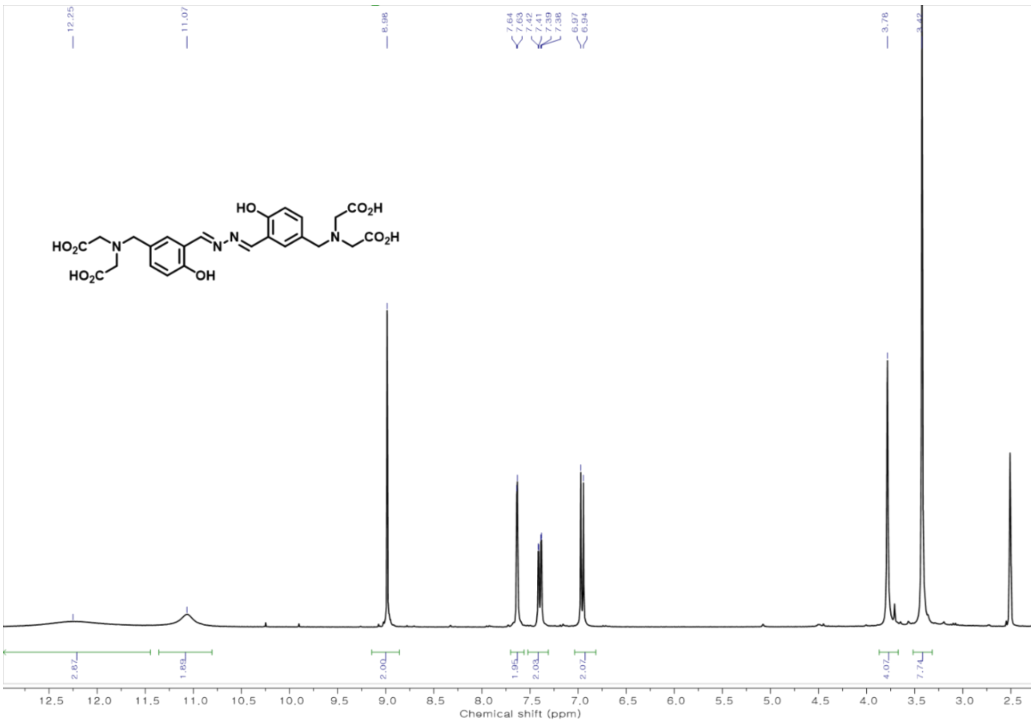

Figure S2. ^{1}H NMR spectrum of compound **1** in DMSO-d₆.

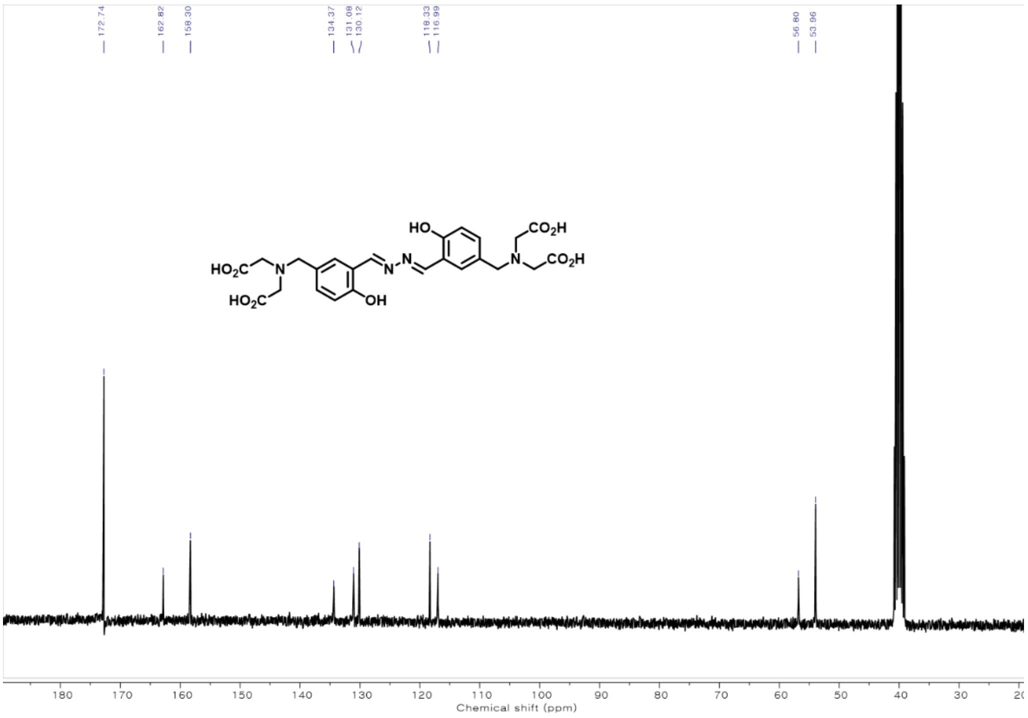

Figure S3. ^{13}C NMR spectrum of compound **1** in DMSO-d₆.

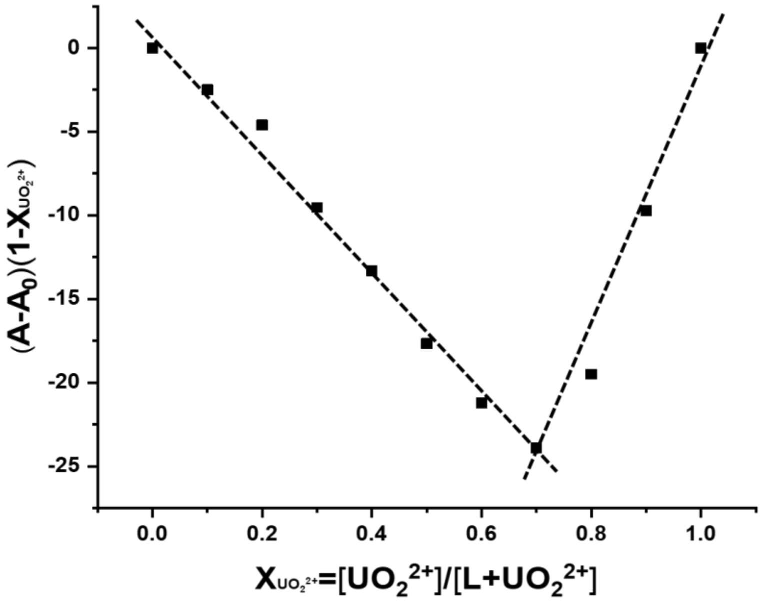

Figure S4. Job's plot for complex formed between compound **1** and UO_2^{2+}.

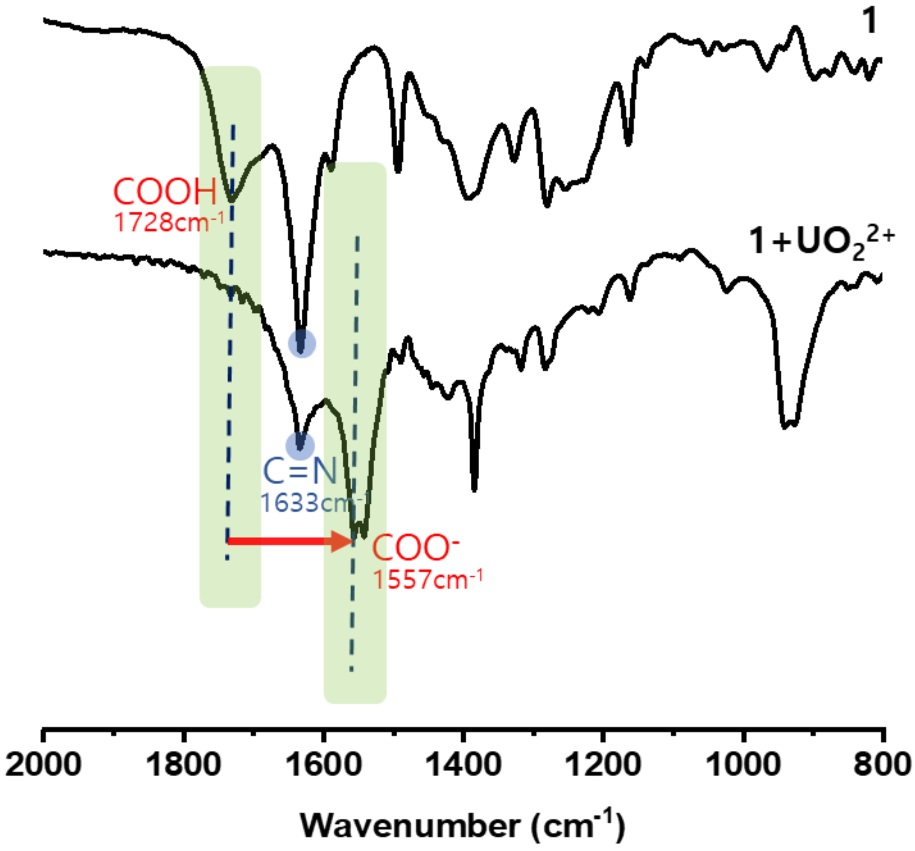

Figure S5. FT-IR spectra of **1** and **1** with UO_2^{2+}.

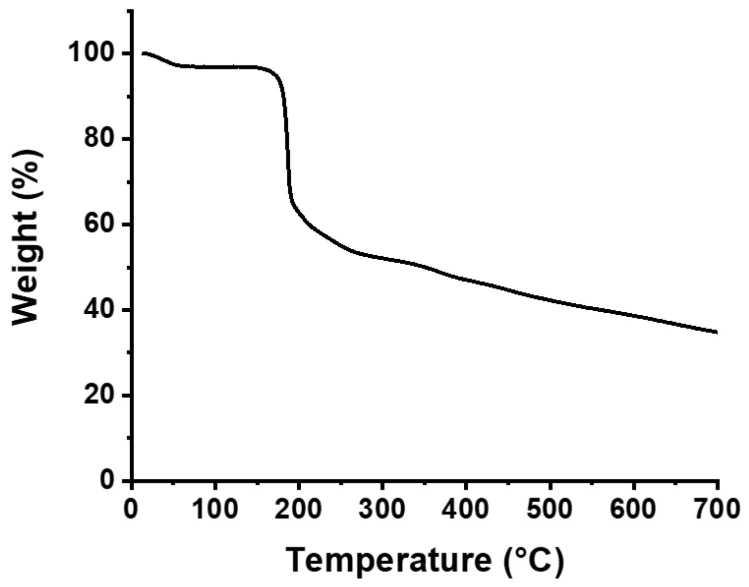

Figure S6. TGA thermogram of compound **1**.

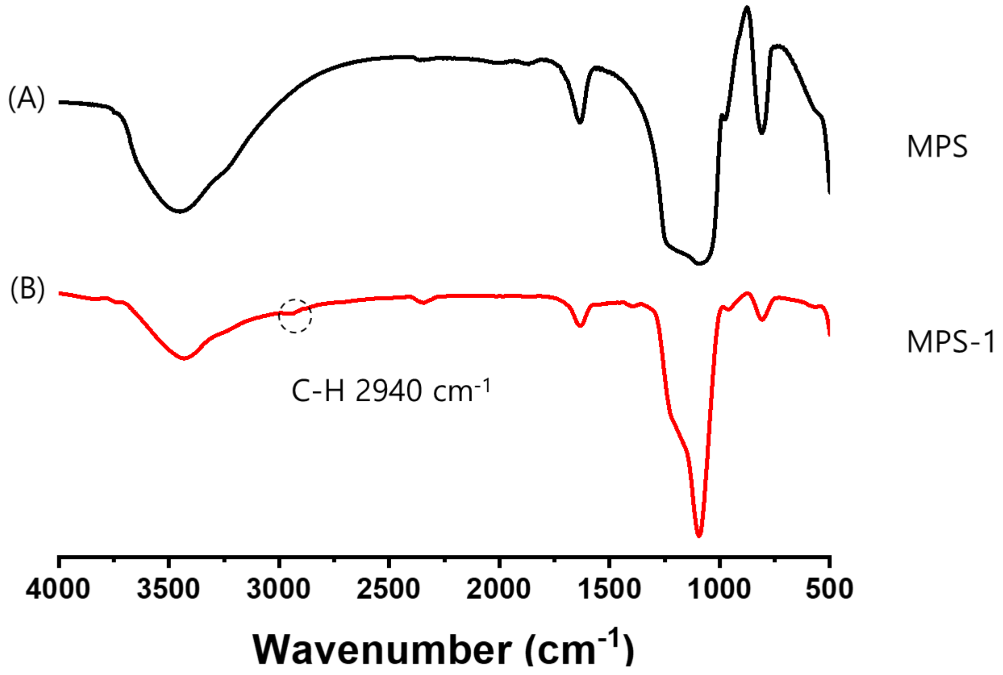

Figure S7. FT-IR spectra of (A) **MPS** and (B) **MPS-1**.

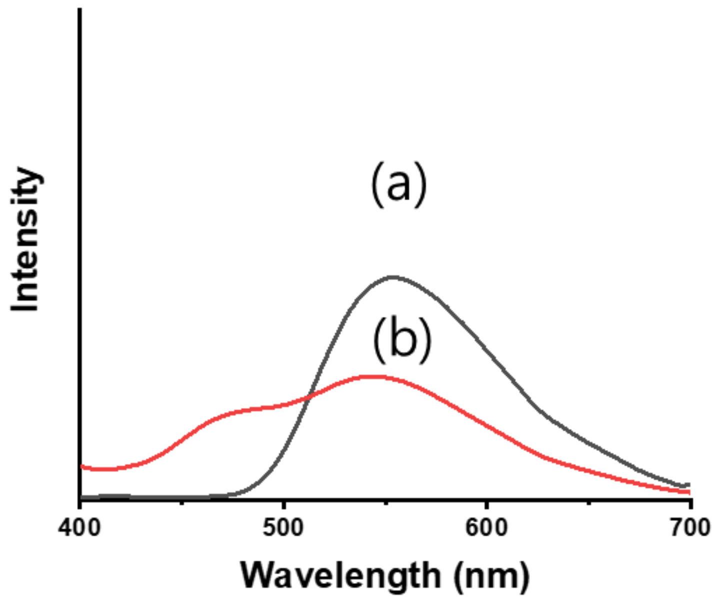

Figure S8. Fluorescence spectra of (a) **MPS-1** (2 mg) in 3.5% NaCl solution (2 mL) and (b) **MPS-1** (2 mg) with UO_2^{2+} solution (100 ppb) in 3.5% NaCl (2 mL).

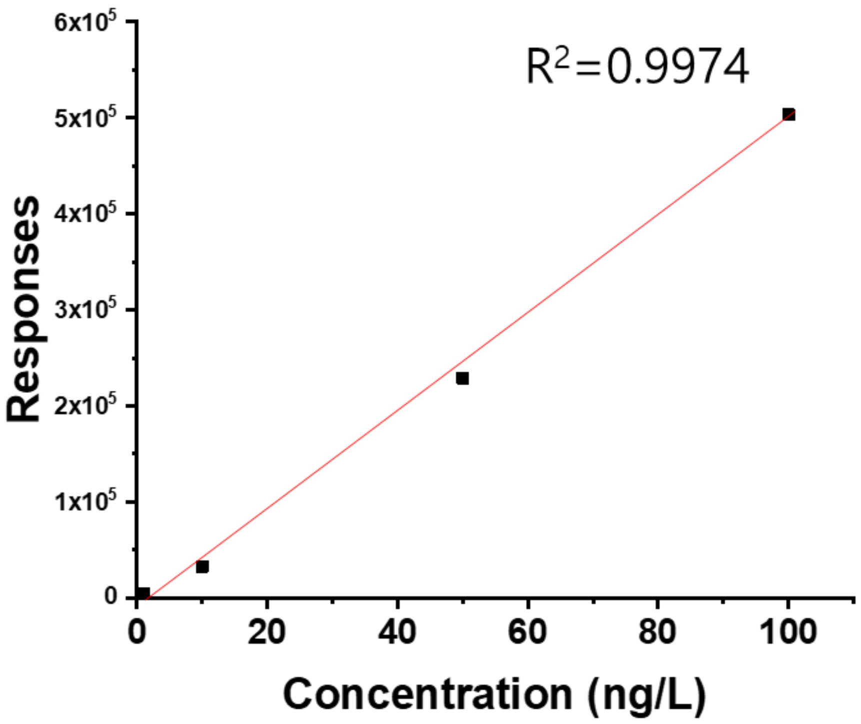

Figure S9. Linear equation of various concentrations of UO_2^{2+}.

Table S1. Adsorption Capacities of **MPS-1** for UO_2^{2+} (100 ppb) solution.[a]

**MPS-1** (mg)	Removal of UO_2^{2+} (%)	SD	RSD(%)
1	69.65	4.14	13.64
3	96.28	0.44	12.02
5	95.16	0.60	12.47
**MPS** (mg)	Removal of UO_2^{2+} (%)	SD	RSD(%)
5	1.85	0.18	9.72

[a]SD = standard deviation; RSD = relative standard deviation.

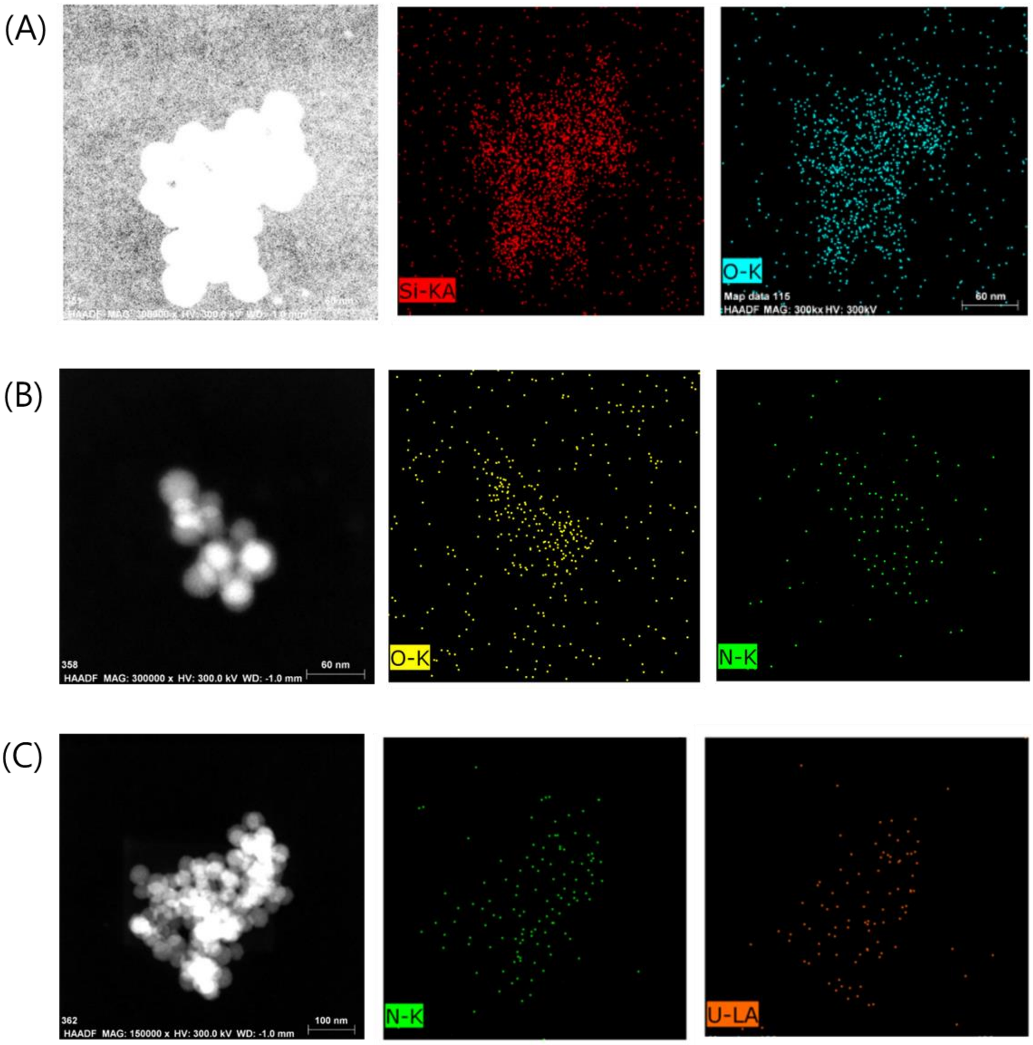

Figure S10. TEM EDX mapping of (A) **MPS** (B) **MPS-1** and (C) **MPS-1** with UO_2^{2+}.

Table S2. Adsorption Capacities of **MPS-1** (5 mg) with various metal ions (100 ppb) solution.

Removal of metal ion (%)								
UO_2^{2+}	Na^{+}	Mg^{2+}	Ca^{2+}	Cu^{2+}	Ag^{+}	Ni^{2+}	Mn^{2+}	Pb^{2+}
95.26	5.21	15.18	42.31	27.54	21.87	23.11	17.59	26.62
